# Pesticide residues in cereal crop grains in Poland in 2013

**DOI:** 10.1007/s10661-015-4566-7

**Published:** 2015-05-07

**Authors:** Elżbieta Malinowska, Kazimierz Jankowski, Jacek Sosnowski, Beata Wiśniewska-Kadżajan

**Affiliations:** Department of Grassland and Landscape Architecture, Siedlce University of Natural Sciences and Humanities, B. Prusa 14 Street, 08 – 110 Siedlce, Poland

**Keywords:** Pesticide residues, Cereals, QuEChERS, Poland

## Abstract

This paper presents the results of the audit on pesticide residues in cereal grains throughout Poland in 2013. The number of all samples of cereal grains was 380. Altogether 292 active substances of plant protection products were checked in the audit. Qualitative and quantative analyses were done according to Polish Standard PN-EN 15562:2008, using the LC-MS/MS technique. The methods (QuEChERS) is based on extraction of residues of plant protection products from a sample using acetonitrile. In the samples analyzed, 62 % of them did not contain any pesticide residues, 34 % of samples of cereal grains contained such residues but below the maximum residue limit, 3 % contained residues over the maximum limit, whereas 1 % of the samples contained illegal substances. The lowest amounts of pesticide residues were found in cereal grains coming from fields with cereal mixtures and in *Avena* grains, while the highest amounts were in *Hordeum* and *Triticum* grains. The substances found most often were fungicide residues. In northern and southern regions of Poland (Silesian, Pomeranian, and Kuyavian-Pomeranian voivodeships), cereal grains with pesticide residues were much more common than in other regions of Poland.

## Introduction

Plant protection products are a wide group of chemicals which are used against plant pests and diseases so effect crop yields can be higher. However, the growth in biomass is not always accompanied by its higher quality. What is more, chemicals used to protect plants can be dangerous to organisms (Kroes et al. 2000, Gorrido et al. 2003, Qian et al. 2010). To maintain plant food safety, Regulation (EC) No 396/2005 on maximum residue levels of pesticides in or on food and feed of plant and animal origin requires the member states of the European Union to monitor pesticide residue levels in food commodities and submit the monitoring results to EFSA and the European Commission (Regulation 2005, Law on food safety and nutrition 2006). After many years of research, it is possible now to assess health hazards caused by plant protection products (Łozowicka et al. 2012). The same research provides means to identify problems and makes inspection of pesticide use more efficient (Regulation 2010). It is also a legal basis for penal sanctions against those breaking rules and regulations.

The aim of this research is to assess occurrence of residues of plant protection products in cereals according to maximum residue limits (MRL).

## Materials and methods

Plant samples from fields were taken randomly by the staff of State Plant Health and Seed Inspection Services in all abovementioned voivodeships. In the research, the following cereals were analyzed: *Hordeum* L. (37 samples), *Triticosecale* (68), *Triticum* (138), *Avena* L. (30), *Secale* L. (50), and mixed cereals (57). Altogether in 2013, there were 380 samples with 292 active substances of plant protection products audited. The Department of Food Safety of the Research Institute of Horticulture in Skierniewice is granted a laboratory accreditation certificate according to Polish Standard PN-EN ISO/IEC 17025:2005. Quality control procedures used there were introduced according to SANCO/12459/2011 Method Validation and Quality Control Procedures for Pesticides Residue Analysis in Food and Feed from 01 January 2012 (Method validation 2012). Quantitative and qualitative analyses were done according to Polish Standard PN-EN 15562:2008, using the liquid chromatography coupled to tandem mass spectrometry (LC-MS/MS) technique (Polish Committee for Standardization 2008). The method is based on extraction of residues of plant protection products from a sample using acetonitrile (QuEChERS), and then, the analysis of those products is done using the liquid chromatograph coupled to tandem mass spectrometer (Drożdżyński and Walorczyk 2010). The QuECHERS method can be modified according to the type of the substance, kinds of matrices tested, equipment used, and analytical technique available in the laboratory (Lehotay et al. 2010). The results of the research on residues of plant protection products in cereal grains are taken from the monitoring of the Research Institute of Horticulture in Skierniewice (Report 2013) (http://www.inhort.pl).

## Results and discussion

**Fig. 1:**
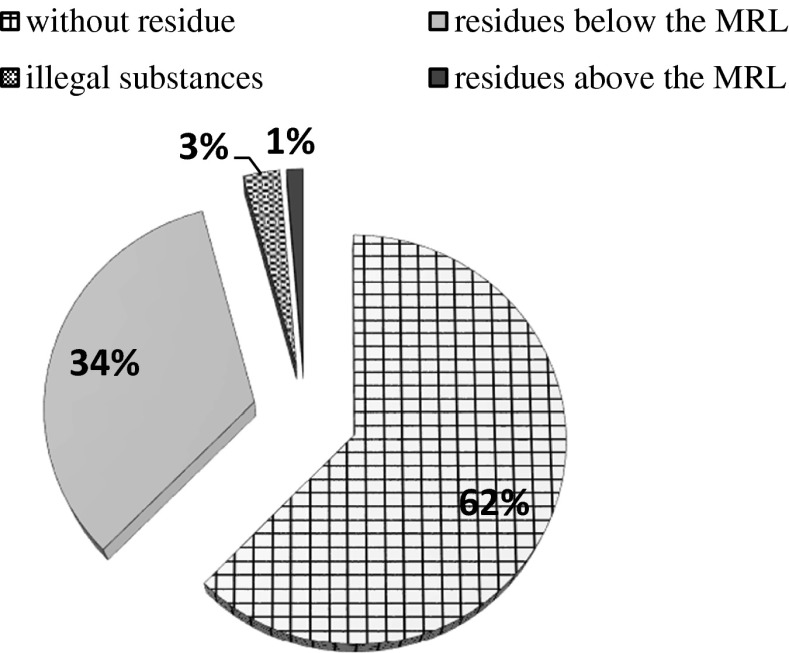
Residues of plant protection products in cereal crop grains throughout Poland in 2013

**Fig. 2:**
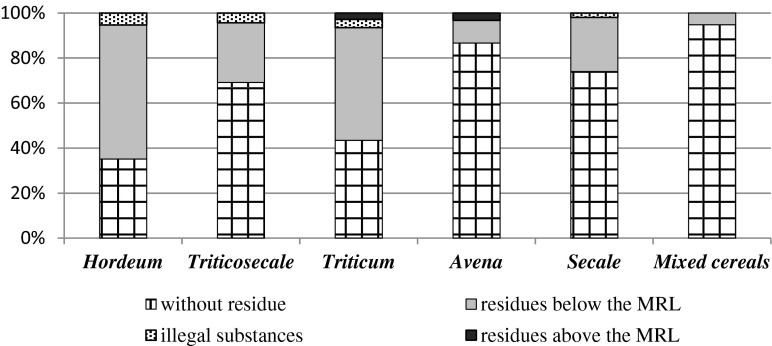
Residues of plant protection products in cereal crop grains in 2013

Out of 380 samples of cereal crops grains, 62 % did not contain any pesticide residues (Fig. 1). While 34 % of samples contained pesticide residues below maximum residue limits, 3 % contained pesticide residues above maximum residue limits and 1 % of samples contained illegal substances. According to some publications (Łozowicka et al. 2012) in Poland between 2008 and 2012, there was a fall in the amount of residues of plant protection products in crops. Comparing to the previous years, in 2012, in particular in northeastern Poland, there was a fall in the percentage of fruit and vegetable samples contaminated with those residues. This percentage was considerably lower than in 2008, while in 2011, it amounted to 33.1 %, in 2010 to 28.3 %, in 2009 to 38.3 %, and in 2008 it was 45.5 %. It proves that the public awareness of the hazards of using chemicals among farmers is growing. Out of the analyzed cereals (Fig. 2), the smallest amount of plant protection products residues was in mixed cereal grains (below 10 %) and *Avena* grains (below 15 %). *Hordeum* and *Triticum* grains contained the highest content of plant protection product residues (over 50 %). There were illegal substances in four types of cereal grains: *Hordeum*, *Triticosecale*, *Triticum*, and *Secale*. Pesticide residues above maximum residue limits were found in *Triticum* grains and *Avena* grains.

In *Hordeum* grains, there were 13 active substances of different chemical composition (Table 1). Those substances were of the following groups: organophosphorus insecticides, carbaminate fungicides, and other fungicides. Out of 37 analyzed *Hordeum* samples, illegal substances (chlorpyrifos 0.02 mg kg^−1^, pencycuron 0.01 mg kg^−1^, procymidone 0.02 mg kg^−1^) were found in three of them. In the case of *Triticosecale*, there were many fewer samples containing plant protection products. Out of 68 *Triticosecale* samples analyzed, there were 21 containing residues of plant protection products. Altogether nine active substances were found including three illegal ones. Those illegal active substances were chlorpyrifos with the concentration of 0.05 mg kg^−1^, propamocarb 0.01 mg kg^−1^, and mandipropamid 0.01 mg kg^−1^. According to some publications (Lu et al. 2000, Figa-Talamanca et al. 2001, Aldrige et al. 2003, Hanke and Jurewicz 2004) they can be mutagenic, carcinogenic and allergenic. Pesticides enter the human body mainly through the digestive system.

**Table 1:** The number of samples and the content of active substances in cereal grains in 2013

Active substances	Number of samples	Without residues	Lower limit of detection (mg kg^−1^)	Maximum residue level (mg^.^kg^−1^)	Number of samples with residues							Number of samples >MRL	MRL (mg kg^−1^)
					0.01	0.02	0.05	0.1	0.2	0.5	1.0		
*Hordeum*													
Chlorpyrifos (N)	37	36	0.005	0.016		1							0.2
Dimethoate	37	36	0.001	0.002	1								0.02
Pirimiphos-methyl	37	30	0.005	0.38		2	3	1		1			5
Carbendazim	37	35	0.001	0.07	1			1					2
Thiophanate methyl	37	36	0.005	0.03			1						0.3
Azoxystrobin	37	36	0.001	0.005	1								0.5
Cyprodinil	37	36	0.005	0.007	1								3
Cyproconazole	37	36	0.005	0.052				1					0.1
Pencycuron (N)	37	36	0.001	0.003	1								0.05
Procymidone (N)	37	36	0.005	0.01		1							0.01
Propiconazole	37	36	0.005	0.019		1							0.2
Spiroxamine	37	36	0.005	0.007	1								0.3
Tebuconazole	37	32	0.005	0.077	1	2	1	1					2
*Triticosecale*													
Chlorpyrifos (N)	68	67	0.005	0.022			1						0.05
Dimethoate	68	65	0.001	0.003	3								0.05
Pirimiphos-methyl	68	62	0.005	0.41	1		2		2	1			5
Carbendazim	68	66	0.001	0.008	2								0.1
Propamocarb (N)	68	66	0.001	0.024	1		1						0.1
Epokskonazol	68	66	0.005	0.012	1	1							0.6
Mandipropamid (N)	68	67	0.001	0.003	1								0.01
Spiroxamine	68	65	0.005	0.002	3								0.05
Tebuconazole	68	67	0.005	0.012		1							0.2
*Triticum*													
Chlorpyrifos (N)	138	137	0.005	0.005	1								0.05
Dimethoate	138	135	0.001	0.009	3								0.05
Pirimiphos-methyl	138	128	0.005	0.64	2	2	2		2	1	1		5
Permethrin (N)	138	134	0.005	0.03	3	1						1I	0.01
Acetamiprid (N)	138	137	0.001	0.001	1								0.03
Phenylphenol (N)	138	136	0.005	0.015	1	1							0.05
Imidacloprid	138	137	0.01	0.011		1							0.1
Carbendazim	138	131	0.001	0.025	5		2						0.1
Propamocarb (N)	138	137	0.005	0.013		1							0.1
Thiophanate methyl	138	137	0.005	0.033			1						0.05
Azoxystrobin	138	131	0.001	0.003	7								0.3
Boscalid	138	137	0.005	0.013		1							0.5
Cyproconazole	138	136	0.005	0.019		2							0.1
Epoxiconazole	138	135	0.005	0.007	3								0.6
Fenpropimorph	138	134	0.001	0.015	2	2							0.5
Pencycuron (N)	138	137	0.001	0.003	1								0.05
Propiconazole	138	137	0.005	0.057				1				1 F	0.05
Spiroxamine	138	131	0.001	0.017	6	1							0.05
Tebuconazole	138	117	0.005	0.790	3	9	7			1	1^a^	1 F	0.2
Chlorpropham (N)	138	137	0.005	0.064				1				1H	0.02
*Avena*													
Pirimiphos-methyl	30	28	0.005	0.25					1	1			5
DEET (N)	30	29	0.005	0.016		1						1I	0.01
Carbendazim	30	29	0.001	0.018		1							2
*Secale*													
Chlorpyrifos (N)	50	49	0.005	0.02			1						0.05
Pirimiphos-methyl	50	47	0.005	0.028	1		2						5
Timetoksam	50	49	0.005	0.007	1								0.005
Carbendazim	50	48	0.001	0.011	1	1							0.1
Thiophanate methyl	50	49	0.005	0.033			1						0.05
Cyproconazole	50	49	0.005	0.02			1						0.1
Epoxiconazole	50	49	0.005	0.007	1								0.6
Propiconazole	50	49	0.005	0.013		1							0.05
Spiroxamine	50	48	0.001	0.002	2								0.05
Mixed cereals													
Pirimiphos-methyl	57	55	0.005	0.039		1	1						5
Carbendazim	57	56	0.001	0.003	1								–

*N* shortage of registered agents for use, *MRL* maximum residue limits, *I* insecticides, *F* fungicides, *H* herbicides

^a^Result relates the whole plant

Even small amounts of them, if they enter the human or animal body for a longer time, may be hazardous to living organisms, in particular to those organisms which are at the end of the food chain (Kroes et al. 2000, Gorrido et al. 2003).

The greatest number of all cereal samples analyzed was those with *Triticum* grains, because this cereal accounts for the greatest share in the whole crop area, about 27 % (Central Statistical Office 2013). In 78 samples, out of 138 tested ones, there were residues of 20 different active substances in *Triticum* grains with seven different illegal substances found in nine samples of those grains. Concentration of two such substances was higher than maximum residue limits, that is permethrin, which is an organophosphorus fungicide, and chlorpropham, which is a herbicide. In three samples with *Triticum* grains, two more substances above maximum residue limits were found. Those were chemicals of the fungicide group, tebuconazole, two times more above the maximum residue limit, and propiconazole, two and five times more above the maximum. In one sample with *Avena* grains, DEET or diethyltoluamide, an insecticide, was found in the amount above the maximum residue limit.

There were no samples with substances over the maximum residue limits in *Secale* grains. Out of 50 analyzed samples of *Secale* grains, 13 contained pesticide residues, including one sample with an illegal substance, which was chlorpyrifos. In 57 mixed grains samples, only three contained pesticide residues but illegal substances and plant protection products over maximum residue limits were not found.

Notifications should be prepared according to Rapid Alert System for Food and Feed 2006 if a chemical is used in a way inconsistent with the recommendations or when the content of the chemical in plant products is over the maximum residue limits. Together with a notification, a procedure is started as well as an assessment of consumers’ health hazards (Kleter et al. 2009). According to RASFF, in 2013, four substances, if found in *Triticum* grains, had to be notified to the EU Commission (permethrin, chlorpropham, propiconazole, and tebuconazole) and one substance had to be notified if found in *Avena* grains (DEET). Besides, illegal substances in all cereal grains, except for mixed cereal crops, should also be notified to RASFF. Podbielska et al. (2013) say that in Poland, illegal substances are quite commonly used in plant protection. Studying vegetable samples cultivated under cover, the authors found the highest amount of pesticide residues in green onion (50 %), pepper (50 %), and tomatoes (45 %). The same research proved the presence of illegal substances used for plant protection—chlorothalonil and chlorpyrifos.

**Fig. 3:**
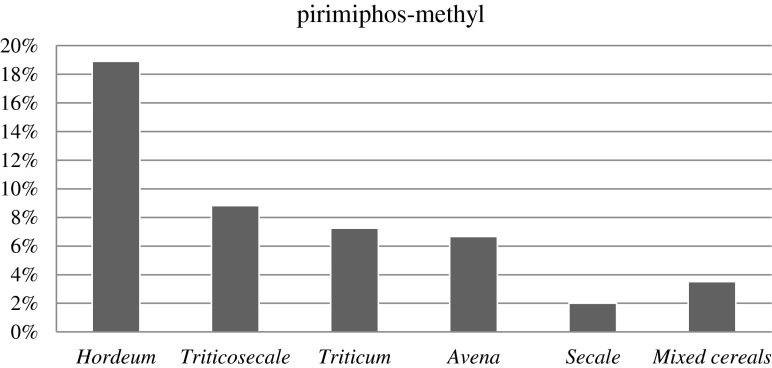
The percentage of organosphosphoric herbicides in cereal grains

Fungicides, like pirimiphos-methyl, spiroxamine, and tebuconazole, and insecticides were the most common substances found in cereal grains (Łozowicka et al. 2012). However, herbicide residues were only found in *Triticum* grains. In all cereal grains tested, residues of organosphosphoric insecticides were found, that is pirimiphos-methyl (Fig. 3). In *Hordeum* grains, the residues of this insecticide were also considerable, with 18.9 %. In the other cereal grains, the amount was quite high: 8.82 % in *Triticosecale*, 7.25 %, in *Triticum* grains, and 6.66 % in *Avena* grains. The smallest amounts were found in *Secale* grain, 2 %, and mixed grains, 3.5 %.

In the research material, different kinds of residues were found with 32 out of 380 samples of cereal grains containing two or more kinds of residues, which accounted for 8.42 % (Table 2). In most cases, those were samples with two active substances (5.8 %). In one sample of barley and one with wheat, there were five active substances.

**Table 2:** Cereal grain samples with high occurrence of residues

Cereal species	Type of substances and content in mg kg^−1^	Voivodeships
*Hordeum*	Tebuconazole (0.01), spiroxamine (0.0066)	LBS
*Hordeum*	Pirimiphos-methyl (0.011), azoxystrobin (0.0045)	KP
*Hordeum*	Dimethoate (0.0017), carbendazim (0.07), tebuconazole (0.077), thiophanate methyl (0.03)	SL
*Hordeum*	Chlorpyrifos (0.016), tebuconazole (0.0076), cyprodinil (0.007), procymidone (0.01), propiconazole (0.019)	LBS
*Triticum*	Pirimiphos-methyl (0.64), carbendazim (0.0014)	LD
*Triticum*	Fenpropimorph (0.015), tebuconazole (0.036)	POD
*Triticum*	Dimethoate (0.0096), pirimiphos-methyl (0.018)	OP
*Triticum*	Azoxystrobin (0.003), cyproconazole (0.01)	DL
*Triticum*	Azoxystrobin (0.028), tebuconazole (0.011)	OP
*Triticum*	Cyproconazole (0.019), tebuconazole (0.031)	OP
*Triticum*	Spiroxamine (0.023), tebuconazole (0.006)	SL
*Triticum*	Pirimiphos-methyl (0.38), carbendazim (0.0027)	SL
*Triticum*	Chlorpyrifos (0.005), phenylphenol (0.005)	POM
*Triticum*	Acetamiprid (0.0013), pencycuron (0.0031)	POM
*Triticum*	Azoxystrobin (0.0033), fenpropimorph (0.0074)	ZP
*Triticum*	Pirimiphos-methyl (0.034), azoxystrobin (0.0017)	KP
*Triticum*	Pirimiphos-methyl (0.023), tebuconazole (0.0056)	POM
*Triticum*	Pirimiphos-methyl (0.4), imidacloprid (0.011)	SL
*Triticum*	Carbendazim (0.038), propamocarb (0.013)	POM
*Triticum*	Boscalid (0.013), spiroxamine (0.017), tebuconazole (0.014)	SL
*Triticum*	Dimethoate (0.028), carbendazim (0.018), tebuconazole (0.039)	DL
*Triticum*	Azoxystrobin (0.024), carbendazim (0.025), tebuconazole (0.024), thiophanate methyl (0.033)	SL
*Triticum*	Epoxiconazole (0.007), permethrin (0.006), pirimiphos-Me (0.005), tebuconazole (0.01)	KP
*Triticum*	Epoxiconazole (0.006), tebuconazole (0.015), permethrin (0.005), pirimiphos-methyl (0.009), carbendazim (0.021)	POM
*Triticosecale*	Mandipropamid (0.0032), propamocarb (0.024)	WLP
*Triticosecale*	Pirimiphos-methyl (0.41), spiroxamine (0.0017)	WLP
*Triticosecale*	Pirimiphos-methyl (0.007), spiroxamine (0.0016)	WLP
*Triticosecale*	Epoxiconazole (0.005), dimethoate (0.0025)	KP
*Triticosecale*	Epoxiconazole (0.012), dimethoate (0.0011), carbendazim (0.0026)	KP
*Secale*	Pirimiphos-methyl (0.023), epoxiconazole (0.007)	SL
*Secale*	Carbendazim (0.0042), thiamethoxam (0.0072), thiophanate methyl (0.033)	LBL
*Secale*	Pirimiphos-methyl (0.009), propiconazole (0.013), cyproconazole (0.02)	POM

Voivodeships: *LBS* Lubusz, *KP* Kuyavian-Pomeranian, *SL* Silesian, *POD* Podlasie, *OP* Opole, *POM* Pomeranian, *ZP* West-Pomeranian, *WLP* Greater Poland, *LBL*-Lublin, *DL* Lower Silesian, *LD* Łódz

There were not many samples with three residues (1.3 %) or four (0.79 %). Because plant protection products have become more effective, they are more commonly used and it contributes to accumulation of those products in plants, despite a low content of active ingredient in the substance, and it leads to the fact that maximum residue limits in food and feed are often exceeded. A breakdown by region showed some differences. The highest occurrence of residues was in the Silesian as well as Pomeranian and Kuyavian-Pomeranian voivodeships. In mideastern Poland (Mazovian, Warmian-Masurian, Lesser Poland, and sub-Carpathian voivodeships), there were no samples with a high content of pesticide residues. A high content of residues, above maximum limits, is related to many factors like weather conditions, the way the spray is carried out, pesticide doses, number of insects, the kind of disease, or whether the withdrawal period is maintained.

## Conclusions

The amount of those residues is closely monitored because they can be harmful to living organisms. Analyzing the results of the audit on pesticide residues in Poland it can be said that out of 380 samples of the cereal grains, 62 % did not contain any residues, 34 % residues of plant protection products were below limits, 3 % were above maximum residue limits, while 1 % of samples contained illegal substances.

Grains of *Avena* and mixed cereals contained the smallest amount of pesticide residues while *Hordeum* and *Triticum* the highest. The most common substances found were fungicides.

Cereal grains with a high occurrence of residues of plant protection products mainly came from the north and south of Poland because of more intensive farming there.
